# The Effects of Smoking and Drinking on Cardiovascular Disease and Risk Factors

**Published:** 2006

**Authors:** Kenneth J. Mukamal

**Affiliations:** Kenneth J. Mukamal, M.D., is associate professor of medicine at the Department of Medicine, Beth Israel Deaconess Medical Center, Harvard Medical School, Boston, Massachusetts

**Keywords:** Alcohol and tobacco, alcohol and other drug (AOD) consumption, smoking, tobacco in any form, comorbidity, risk factors, beneficial moderate alcohol consumption, risk and protective factors, cardiovascular disorder, stroke, coronary artery disorder, congestive heart failure, high blood pressure, hypertension, cholesterol, alcoholic cardiomyopathy

## Abstract

Research on how tobacco and alcohol use interact to influence risk for cardiovascular disease is limited. Alcohol consumption of three or more drinks per day and cigarette smoking share similar, and probably additive, effects on some forms of cardiovascular disease. There is relatively little evidence, however, that the effects are worse when smoking and drinking occur together than would be expected from their independent effects. In most cases, moderate drinking does not share these risks and even has opposite effects of cigarette smoking on some risk factors. Ongoing public health efforts to minimize tobacco use and harmful drinking should result in clear and important gains to the nation’s cardiovascular well-being.

An extraordinary body of research has sought to understand the links between smoking cigarettes and drinking alcohol, and an equally substantial body of evidence has demonstrated their synergy in causing cancer, birth defects, and other medical problems. In contrast, there generally has been little evidence that they interact to influence cardiovascular disease. Indeed, one recent review found only a single article that identified a probable interaction between alcohol and tobacco on risk of heart disease resulting from narrowing of the arteries that supply blood and oxygen to the heart (i.e., coronary heart disease) (Taylor and Rehm 2006). This article seeks to examine more broadly the ways that tobacco and alcohol may jointly affect risk of cardiovascular disease.

## A Complicated Issue

Several factors complicate the interactions between tobacco and alcohol on cardiovascular disease. First, dose matters. The relationship between smoking and risk of cardiovascular disease is dose dependent—more tobacco leads to more disease. For alcohol consumption, however, the issue is more complex. Most evidence suggests that consumption in the range of 3 to 14 drinks per week is associated with lower risk of heart attack (i.e., myocardial infarction) and possibly of other forms of cardiovascular disease, such as blockage in an artery that supplies blood to the brain, resulting in a deficiency in blood flow (i.e., ischemic stroke) or failure of the heart to pump blood sufficiently throughout the body (i.e., congestive heart failure). However, intake of three or more drinks per day clearly increases the risk of ischemic stroke, and heavier drinking may well increase the risk of myocardial infarction.

Second, cardiovascular disease encompasses a variety of conditions with a diverse set of causes or origins. Smoking is clearly linked to a higher risk of nearly all forms of cardiovascular disease, including myocardial infarction, ischemic stroke and bleeding into the brain (i.e., hemorrhagic stroke), congestive heart failure, and narrowing of the arteries in the extremities (i.e., peripheral arterial disease) (Burns 2003). The relationship between alcohol use and cardiovascular risk factors is not so clear. Moderate drinking has been associated with a consistently lower risk of myocardial infarction, but only a modestly lower risk of ischemic stroke, and a higher risk of hemorrhagic stroke. Simply combining all of these conditions together as “cardiovascular disease” will tend to blur these distinctions.

Third, even those types of cardiovascular disease directly linked to the gradual build-up of fatty deposits (i.e., plaques) in the arteries, such as myocardial infarction and stroke, represent acute events superimposed on the background process of the gradual narrowing and hardening of the arteries (i.e., atherosclerosis). Atherosclerosis itself is dynamic and involves cholesterol transport into and out of cells in the blood vessel wall, the entry of inflammatory cells, and abnormal function of the cells lining the vessel surface (i.e., endothelial cells). The final trigger in this pathway is often a blood clot that forms at the site of a plaque whose cap has ruptured, exposing the blood to irritants within the plaque. Tobacco and alcohol use may have chronic effects on several steps in the gradual atherosclerotic process and more acute effects on the formation of blood clots that often trigger actual clinical events.

Fourth, and perhaps most difficult to address, is the nature of the relationship between alcohol consumption and cigarette smoking and how it bears on our understanding of each. In both experimental and observational studies of alcohol consumption and cardiovascular disease, cigarette smoking is treated as a confounder or nuisance parameter. That is, researchers recognize that smoking is common among drinkers and that it is a strong risk factor for heart disease that could cloud the true effect of alcohol consumption. Investigators typically study the effect of alcohol independent of smoking, either by mathematical adjustment or by examining smokers and nonsmokers separately. This approach implicitly views smoking and drinking as shared consequences of specific lifestyle or behavior patterns and ignores the possibility that alcohol consumption itself makes individuals more likely to smoke cigarettes (Shiffman and Balabanis 1995). If alcohol consumption itself leads to cigarette smoking to even some degree, then understanding the full effects of alcohol will require accounting for a difficult complexity—cigarette smoking among alcohol drinkers may be related both to shared lifestyle habits and to direct effects of alcohol. This complexity is equally important for studies of tobacco use, as they need to incorporate the possibility that smoking may lead to alcohol consumption (Madden et al. 2000). This issue will challenge researchers for the foreseeable future.

## An Important Public Health Issue

Potential relationships of alcohol use and smoking on cardiovascular disease are of great public health importance. The American Heart Association (2005) estimates that in 2003, over 71 million Americans had some form of cardiovascular disease, representing more than 34 percent of the United States population. In 2002, cardiovascular disease caused or contributed to more than 1.4 million deaths in the United States, representing about 58 percent of all mortality. Tobacco use is an important contributor to this burden. About 21 percent of adult Americans reported using tobacco in 2004. Although tobacco use rates generally have declined over the last 40 years, some 4,000 individuals become new regular smokers every day. Given that more than 85 percent of smokers drink alcohol, and that drinkers are 75 percent more likely to smoke than are abstainers, the public health ramifications of joint use of alcohol and tobacco may be substantial indeed.

## Effects on Cardiovascular Risk Factors

Alcohol and tobacco use both have important effects on cardiovascular risk factors. Overall, the two generally do not affect the same risk factors in the same way, although levels of blood pressure and triglycerides (i.e., fats in the blood) may be important exceptions.

**Table:** Proposed Qualitative Relationships of Light to Moderate or Heavier Alcohol Consumption (Relative to Abstention) and Cigarette Smoking to Cardiovascular Disease and Its Risk Factors

	Light to Moderate Alcohol Intake	Heavier Alcohol Intake	Cigarette Smoking
	
Cardiovascular Disease			
Myocardial Infarction	↓	↑	↑↑
Ischemic Stroke	↔	↑↑	↑↑
Hemorrhagic Stroke	↑	↑↑	↑↑
Congestive Heart Failure	↓	↑↑	↑↑
Cardiovascular Risk Factors			
HDL-C	↑↑	↑↑	↓
Triglycerides	↑	↑↑	↑↑
Blood Pressure	↔	↑↑	↑
CRP	↓	↔	↑↑
Platelet Function	↓	↓ ↓	↑↑

Alcohol consumption of three or more drinks per day clearly raises blood pressure, one of the most important cardiovascular risk factors (Klatsky 1996). As a result, consumers of three to five drinks per day have a roughly 50 percent higher risk of high blood pressure (i.e., hypertension); risk increases even more with heavier intake. Lighter intake, however, has generally not been associated with blood pressure and, in a few studies, has actually been associated with a modestly lower risk of hypertension (Thadhani et al. 2002). The relationship between smoking and blood pressure is less clear, in part because smokers tend to be leaner than nonsmokers. However, in some laboratory studies and well-controlled population studies, smoking appeared to raise blood pressure or risk of hypertension to a modest degree (Niskanen et al. 2004).

There are similar relationships between alcohol and tobacco use and levels of triglycerides, a fat in the blood that has been linked to risk of coronary heart disease in some studies. Alcohol intake has long been known to increase triglyceride levels, apparently in a dose-dependent manner (Rimm et al. 1999). Interestingly, many clinical trials of alcohol consumption have documented this increase in triglyceride levels, but it may pertain only to men. Some recent trials among women have surprisingly found that moderate consumption may reduce triglyceride levels (Davies et al. 2002). Cigarette smoking also increases triglyceride levels, and studies of young adults have identified early use of alcohol and tobacco as key determinants of subsequent levels of serum triglycerides (Croft et al. 1987).

The other fat (i.e., lipid) most closely associated with alcohol intake is high-density lipoprotein cholesterol (HDL-C), which increases with greater alcohol intake until fairly high levels of consumption. HDL-C is involved with reverse cholesterol transport, the process of returning cholesterol from the peripheral tissues back to the liver for disposal, and higher levels of HDL-C are very strongly related to lower risk of myocardial infarction. Given the connections of alcohol and HDL-C, it is thought that HDL-C accounts for about half of the apparent benefit of alcohol consumption on the risk of cardiovascular disease. Although alcohol intake is the lifestyle factor most closely correlated with HDL-C levels at a population level, smoking also is correlated with HDL-C but in the opposite direction (Ellison et al. 2004).

Several other cardiovascular risk factors are affected by smoking and drinking, often in opposite ways. For example, alcohol consumption, both in moderation and at excessive levels, tends to inhibit the activity of platelets, the blood cells that form clots, and to reduce levels of fibrinogen, a blood protein involved in clotting. This blood “thinning” may explain why even moderate drinking can increase the risk of certain bleeding complications, such as hemorrhagic strokes, while lowering the risk of heart attacks and other diseases characterized by blood clots. In contrast, cigarette smoking activates platelets and renders them more likely to form clots. In a somewhat similar fashion, moderate drinking has been linked to lower levels, and heavier drinking to higher levels, of C-reactive protein (CRP) (Imhof et al. 2001), a marker of inflammation in the body, whereas cigarette smoking consistently appears to increase CRP levels.

## Effects on Risk of Cardiovascular Disease

As noted above, there are many types of cardiovascular disease. This section will review the effects of alcohol and tobacco use on three of the most common manifestations of clinical cardiovascular disease—coronary heart disease, stroke, and congestive heart failure.

Alcohol intake in the range of 3 to 14 drinks per week consistently has been associated with decreased risk of myocardial infarction in observational studies, both among men and women and in a variety of countries (Corrao et al. 2000). In nearly all studies, this association has been similar among smokers and nonsmokers, suggesting that although smoking clearly increases coronary risk two- to four-fold (American Heart Association 2005), alcohol acts similarly whether or not an individual smokes. Even the few studies that have identified apparent smoking-related differences in how alcohol use is associated with coronary heart disease do not agree on whether smokers or nonsmokers are most likely to demonstrate the lower coronary risk linked to moderate drinking. However, few of these population studies enrolled sufficient numbers of heavy drinkers to understand their risk of myocardial infarction with certainty or to examine how heavy drinking might interact with smoking. In addition, the magnitude of risk related to smoking is far larger than any ostensible benefit related to moderate drinking, so even those current smokers who drink moderately remain at high risk for myocardial infarction.

Another manifestation of coronary heart disease is angina, or chest pain related to an imbalance between oxygen need and oxygen delivery to the heart muscle, especially during exercise. Interestingly, heavy alcohol consumption acutely appears to worsen this imbalance and shortens the amount of time that a person can exercise before signs of oxygen deficiency (i.e., ischemia) occur (Rossinen et al. 1996). Likewise, cigarette smoking acutely decreases blood flow and decreases the amount of time a person can exercise before the onset of angina (Deanfield et al. 1986). Alcohol and cigarette smoking also have additive effects on heart rate and blood pressure (Benowitz et al. 1986).

Stroke occurs when blood flow to the brain is acutely interrupted by local occlusion of blood vessels in the brain, dislocation of blood clots elsewhere that then lodge in the brain, or blood vessel rupture. Both regular alcohol intake of three or more drinks per day and cigarette smoking are strong, unequivocal risk factors for ischemic stroke (the most common type) (Goldstein et al. 2006), although little evidence exists that the combination raises risk more than expected from their independent effects. In addition, intake of three or more drinks appears to raise the risk of stroke acutely for at least 24 hours afterward (Hillbom et al. 1999). Even moderate drinking and cigarette smoking also increase the risk for hemorrhagic stroke, a very common type of stroke in Asia. Finally, light drinking has been associated with a lower risk of ischemic stroke than abstention in both men and women (Reynolds et al. 2003), but even this association has undergone revision in recent years. Although older studies initially suggested lowered risk of ischemic stroke among moderate drinkers of a magnitude comparable to the association seen with myocardial infarction, more recent, better designed studies have established that consuming even one drink per day is not associated with lower risk and that intake of one to six drinks per week is likely to be associated with only a modestly lower risk (Reynolds et al. 2003).

The most rapidly increasing form of cardiovascular disease is congestive heart failure, a syndrome in which pressure and fluid accumulate in the lungs because the heart is unable to generate sufficient output, either because of weakened heart muscle (e.g., after a myocardial infarction or from direct heart muscle toxins or infections) or muscle thickening that prevents the heart from filling normally. Alcohol consumed to excess over several years can produce an alcoholic cardiomyopathy, in which alcohol acts as a toxin to weaken the heart muscle directly and hence may improve with abstention. Cigarette smoking also is a strong risk factor for congestive heart failure in the general population (Klatsky et al. 2005), and research with dogs has shown that oral nicotine administration increases the degree of scarring that accompanies alcoholic cardiomyopathy (Rajiyah et al. 1996).

However, more than 20 years ago, Greenberg and colleagues (1982) showed that even the consumption of four to five drinks leads to relaxation of the peripheral blood vessels and could potentially “unload” the failing heart. Subsequently, population-based studies have shown that alcohol intake up to one to two drinks per day might be associated with a lower risk of congestive heart failure (Klatsky et al. 2005). Although this association is partly explained by the lower risk of myocardial infarction linked to moderate drinking, it persisted even among those cases of heart failure that did not appear related to previous myocardial infarction. This association, like that of moderate drinking with myocardial infarction, also appears to be similar among smokers and nonsmokers.

## Lessons from the Russian “Natural Experiment”

Despite the limited evidence that alcohol consumption and tobacco use interact directly to cause or exacerbate cardiovascular disease, the example of the Russian transition from Soviet State to independent nation hints at the extraordinary harm that these substances can potentially produce in combination when consumed in excess, at least during periods of social upheaval (Notzon et al. 1998). Between 1990 and 1994, male life expectancy in Russia declined by an almost unimaginable 6.1 years, coinciding with a 35.7 percent increase in overall mortality rates. Although information on cause of death often is incorrect, alcohol-related causes and injuries alone appeared to account for 29.4 percent of this increase, while heart disease and stroke accounted for another 33.4 percent. Both alcohol use and tobacco imports rose sharply during that period, while other aspects of diet did not clearly deteriorate (i.e., nutritious food did not necessarily become less available). The juxtaposition of the steep increases in alcohol and tobacco use with the marked increase in cardiovascular mortality provides circumstantial evidence that, at least at the extremes, these two substances may interact to precipitate cardiovascular disease with alarming rapidity.

## Conclusions

In summary, alcohol consumption and tobacco use have been associated with a wide variety of cardiovascular diseases, although these associations include both detrimental and (at least for moderate drinking) some potentially beneficial effects. Alcohol intake of three or more drinks per day and cigarette smoking share similar, and probably additive, adverse effects on some forms of cardiovascular disease. Examples of these adverse effects include increases in blood pressure and levels of triglycerides in the blood and higher risks of stroke and congestive heart failure. On the other hand, there is relatively little evidence that the two act synergistically or that the effects are worse when smoking and drinking occur together than would be expected from their independent effects. In most cases, more moderate drinking does not share these risks and even has effects opposite those of cigarette smoking on HDL-C and blood clotting. Nonetheless, because alcohol and tobacco are used together and in excess so commonly, their joint effects are encountered widely throughout the U.S. population. Ongoing public health efforts to minimize tobacco use and harmful drinking should result in clear and important gains to the nation’s cardiovascular well-being.
